# Visiting Sick People: Is It Really Detrimental to Our Health?

**DOI:** 10.1371/journal.pone.0002299

**Published:** 2008-06-04

**Authors:** David Fouchet, John O'Brien, Dominique Pontier

**Affiliations:** 1 UMR-CNRS 5558, Biométrie et Biologie Evolutive, Université de Lyon, Lyon, France; 2 Zoology Department, Trinity College Dublin, Dublin, Ireland; London School of Hygiene & Tropical Medicine, United Kingdom

## Abstract

Intuitively, keeping ones distance from a source of infection would appear to be the best way to limit the occurrence of disease. However, this overlooks the importance of repeated infections in maintaining efficient immune defenses. When acquired immunity has partly waned, re-exposure to the pathogenic agent may lead to mild disease that boosts the immune system. This prevents the total loss of immunity that would lead to classical disease in cases of re-infection. Here, using a mathematical model, we show that avoiding the pathogenic agent is detrimental in some situations, e.g. for pathogens that are highly transmissible, are not excessively lethal and that induce rapidly waning immunity. Reducing exposure to pathogenic agents is among the objectives of most, if not all, public health measures. A better understanding of the factors influencing the severity of a disease is required before applying measures that reduce the circulation of pathogenic agents.

## Introduction

Throughout history, people have fled from sources of infection in an effort to prevent themselves from contracting disease. The enforced isolation of lepers and tuberculosis sufferers and the abandonment of villages infected with Black Death are classic examples of this behaviour. Even today, who among us does not flinch when someone sneezes or coughs beside us?

Theoretically, limiting exposure to pathogens, e.g. by avoiding contact with sick people, should reduce disease occurrence. Implicitly, this assumes that successful attacks by pathogens are always deleterious for the host. But this assumption does not always hold true. For example, the frequency of infections by VZV, the virus responsible for Varicella and Zoster (when the virus naturally reactivates after a latency period), is inversely correlated with the occurrence of Zoster in elderly people [1]. In this case, frequent exposure to the virus boosts the immune system without causing harm and prevents the reactivation of the virus. Similarly, immunity acquired after vaccination [2]–[5], but also after natural infection [3], [6], wanes with time and frequent natural and attenuated re-infections help retain efficient immune defenses [7]–[11]. In these contexts, avoidance of the infectious agent is not always beneficial.

In a previous modelling framework, Aguas et al. [11] illustrated this concept with a mathematical model of pertussis. They argued for the existence of a re-infection threshold [12] above which mild re-infections are frequent and immunity is boosted before waning. Above the threshold, a reduction in the transmission rate of the disease prevented natural boosting of the immune system and, consequently, an increase in the number of severe cases was apparent. This increase in the number of severe cases was coincident with a huge increase in the number of mild infections: an increase in the transmission rate of the disease by around 60% led to a decrease of 20% in cases of severe disease, and a ten-fold increase in the number of mild infections. Even if by definition mild infections cause little harm to their host, one should remember that what Aguas et al. [11] termed a mild infection was in fact an infection that occurred during the period of partial immune protection. While these infections are generally mild, they can sometimes display severe symptoms, e.g. in malnourished or immuno-compromised individuals. Similar results have recently been obtained with a model representing the spread of Malaria [13], a disease for which regular re-infections help to maintain an efficient immune response [10]. The model was compared with clinical data and explained the observed peaks in malaria hospital admissions in children of less than 10 years of age at intermediate transmission rates [14], [15].

In the present paper, we explore the conditions under which avoidance of exposure to the pathogen is detrimental to the host. Waning immunity in the absence of natural boosting is a common phenomenon that may apply to many host-pathogen interactions. Empirical analyses of the durations of immunity and rates of boosting are complex. As an alternative, here we develop a mathematical model to determine the types of diseases for which reducing the transmission rate of the pathogen could lead to the most pronounced adverse effects. Considering individuals with a given rate of exposure to a given pathogen, is it beneficial for them to decrease this rate of exposure? The model helps to define the characteristics of diseases for which classical health measures, which consist of reducing peoples' exposure to pathogenic agents, could fail and even worsen the impact of the pathogenic agent.

## Materials and Methods

### The mathematical model

#### Model with constant exposure rate

In the model we assume that each individual follows one of the two following strategies. Firstly, one can try to reduce their level of exposure to the pathogen, for example by avoiding contact with sick people. We define individuals that follow this strategy as “avoiders”. Secondly, one may take no special precautions to avoid infections and allow the pathogenic agent to infect them normally. Here, we refer to these individuals as “normal”. “Avoiders” are less often infected compared to “normal” individuals, but cannot avoid all infections. We denote *φ* as the relative rate at which “avoiders” become infected compared to “normal” individuals.

To investigate the effect of different levels of infection rate and the duration of immunity on the benefit of avoiding exposure to pathogens, we modified the classical susceptible-infected-recovered (*SIR*) model to integrate different levels of acquired immunity and the boosting effect of attenuated re-infections when it occurs before the level of immunity falls below the protective threshold.

**Figure 1:**
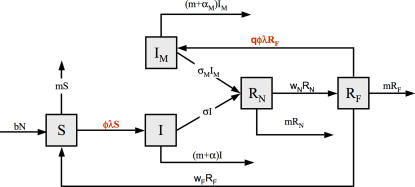
Flow diagram of the five classes of the modified *SIR* model. Arrows represent the transitions, with their associated rates. Transition rates in red are the only ones that differ between “avoiders” and “normal” individuals.

We add one class to the classical *SIR* model (Fig. 1) representing mildly infected individuals (*I_M_*). We also split the *R* class into two: newly recovered individuals (*R_N_*) that are fully protected against re-infection; and formerly recovered individuals (*R_F_*) that can become re-infected and develop the mild form of the disease. Individuals are susceptible at birth, then become infected and develop the classical form of the disease. Subsequent re-infections are attenuated when they occur in the *R_F_* class and lead to a level of immunity as high as that following classical infection (note that this assumption does not modify the main results of the model). Without attenuated infection, individuals lose their immune defense and become fully susceptible.

As a first step we assume a constant rate of infection by the pathogenic agent for each individual. The purpose here is not to understand how the infection rate evolves with pathogen circulation, but only to determine the best strategy for one individual suffering a given (and constant) pathogen exposure. Individuals that choose the “normal” strategy get infected with a constant rate *λ*. “Avoiders” get infected with a lower rate *φλ*, where *φ* is a constant (*φ*<1) that describes how avoidance of the pathogenic agent reduces the frequency of infections. *R_F_* individuals can become mildly infected at a rate *q* times (0<*q*≤1) that of the susceptible individuals having the same behaviour. We assume a constant influx of births (*b*) and a constant natural (i.e. from any cause other than the pathogen) death rate (*m*). Classically and mildly infected individuals recover from the disease at rates σ and σ*_M_* respectively, and die from the disease at rates α and α*_M_* respectively. We call *CM* and *CM_M_* the case mortalities, i.e. the probability that infected individuals die from the infection instead of recovering, for the severe and the mild infections, respectively (*CM* = α/[α+σ] and *CM_M_* = α*_M_*/[α*_M_*+σ*_M_*]). Newly and formerly recovered individuals lose their protection at a rate *w_N_* and *w_F_* respectively.

The model is described by the set of equations (by setting *φ* = 1: we obtain the model for “normal” individuals. *φ*<1: corresponds to “avoiders”):
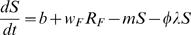


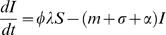


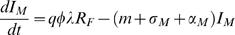


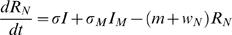


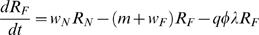



The set of assumptions we have made has allowed us to produce a linear, and therefore simple, mathematical model. From a biological point of view this means that in our model each individual is not affected by the infectious status of other individuals. This implies that we can run the model initially with only “normal” individuals, and then only with “avoiders” and compare how the different strategies alter the impact of the pathogens. Results would be exactly the same if we assume a mixed population with both “avoiders” and “normal” individuals.

Another interesting implication of the linearity of the model is that the rate of influx of newborns (*b*), and thus the host population size, will not have any effect on the results of the model presented here.

#### Model where exposure depends on infected individuals

In the previous section we assumed that the rate of infection of individuals is constant in the population. In fact, for transmissible diseases, the number of infected individuals in the host population will affect the rate of infection of susceptible individuals. When a large proportion of the population avoids infections, the rate of infection of susceptible individuals will be reduced, which can have important consequences on whether or not avoiding infections is a good strategy. To determine the consequences of mass avoidance of infectious agents it is important to incorporate the effect of avoidance on the rate of infection.

This model is similar to that from the previous section, except that now the rate of infection depends on the number of infected individuals. The proportion of individuals avoiding the infection is then a critical factor, so we can no longer assume that the two strategies are independent. Each class *X* is divided into two subclasses, *X^N^* and *X^A^*, which represent the number of individuals in class *X* following the “normal” and “avoider” strategies, respectively. The model then reads:
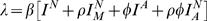


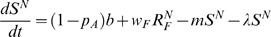


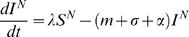


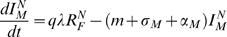


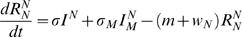


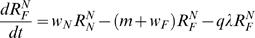


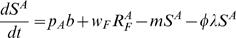


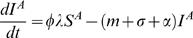


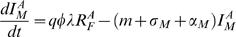


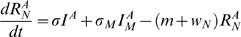


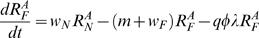
where β is the transmission rate of the pathogen from severely infected “normal” individuals to susceptible “normal” individuals and ρ is the relative rate at which mildly infected individuals transmit the pathogen compared to severely infected ones. To simplify, we assume that “avoiders” are φ times less exposed (φ<1) than “normal” individuals. We also assume that when they are sick, “avoiders” avoid transmitting the pathogen to other individuals and are thus φ times less infectious than “normal” individuals. Furthermore, we assume that mildly infected individuals can also be avoided and, in turn, can avoid transmitting the pathogen with the same success as severely infected individuals. The reality is more complex, since in some cases mild infection may be hard to detect, whereas in other circumstances mild symptoms, such as coughing or sneezing, can be easily detected. For our analysis, this assumption has only a slight qualitative impact on the results presented in this paper.

Note the basic reproductive number for the pathogen in this model is:
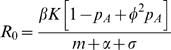
where *K* = *b/m* is the carrying capacity of the host population, i.e. the size of the host population without pathogen. Note that in this case a population consisting only of “avoiders” (*p_A_* = 1) has a 
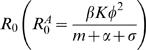
 that is φ*^2^* that of the *R_0_* of a population comprised exclusively of “normal” individuals (*p_A_* = 0, 
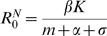
).

#### Parameters

The model is parameterized to represent a typical European or North American human population. Individuals have a 75-year life expectancy (*m* = 1/75, the time unit is years). Both forms of the disease (classical and attenuated) last, on average, two weeks (α+σ = α*_M_*+σ*_M_* = 24). Beyond reducing the severity of the disease, partial immunity also reduces by a factor of ten the probability of becoming infected (*q* = 0.1). In the model with constant exposure rate, “avoiders” are 10 times less often infected than “normal” individuals (φ = 0.1); whereas in the model where the exposure rate depends on infected individuals, we assume that a population made up only of “avoiders” have a basic reproductive number that is 10 times lower than that of a population exclusively composed of “normal” individuals (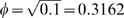). To simplify, we assume that the two phases of immunity, corresponding to full and partial protection, last on overage the same duration, which is 6 months as a basic value (*w_R_* = *w_F_* = 2, if not allowed to vary). The rates λ and β that describe the force of infection of the pathogen in the models are variable in all the situations tested and so have no basic value.

**Figure 2:**
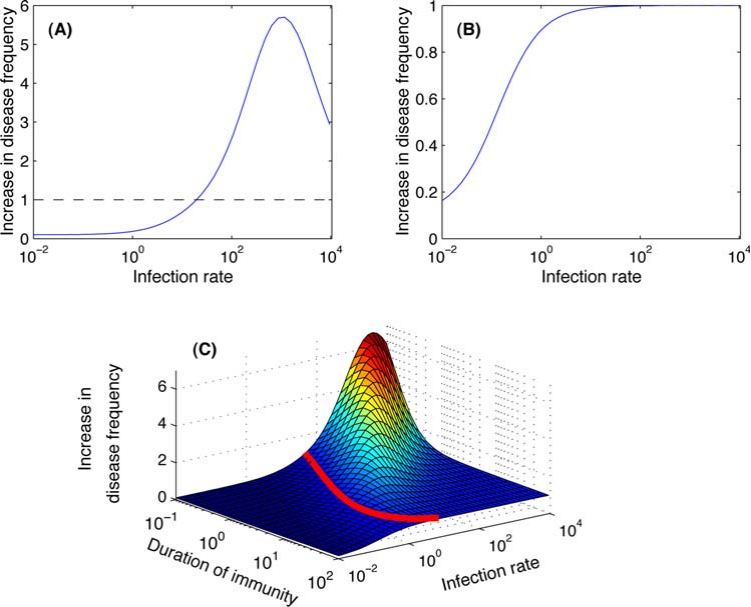
Detrimental effect of pathogen avoidance for non-lethal diseases. It is represented by the relative frequency (*r*, Y-axis) at which “avoider” individuals suffer classical infections compared to “normal” ones (when values are below one, avoiding the pathogenic agent is beneficial), according to the rate of infection by a pathogenic agent (X-axis). (A) For total immune periods of one year, avoiding the pathogenic agent is beneficial only for low infection rates. For high infection rates, both strategies tend to become equivalent since boosts to the immune system are almost systematic; (B) for lifelong immunity, avoiding the pathogenic agent is always a good strategy; and (C) considering a continuum in the total duration of immunity (in years) shows that “avoiders” can be more than six times more at risk of becoming sick compared to “normal” individuals. The threshold where both strategies are equivalent (*r* = 1) is represented with a dashed line (A) or with a bold red line (C).

**Figure 3:**
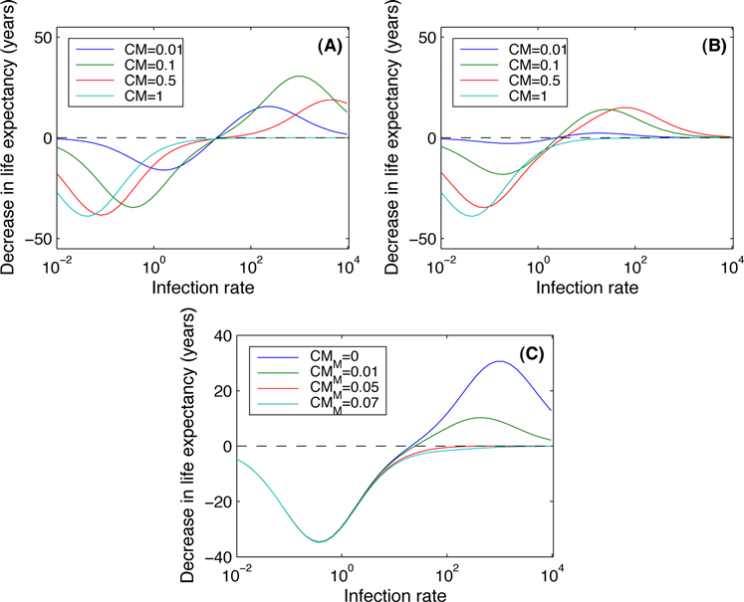
Detrimental effect of pathogen avoidance for lethal diseases. It is represented by the difference in life-expectancy between “normal” individuals and “avoiders” (Y-axis; where it is negative, i.e. below the dashed line, avoiding the pathogenic agent is beneficial), according to the rate of infection by a pathogenic agent (X-axis). (A) For total immune periods of one year, no additional mortality during mild infection and different values for the case mortality of the classical disease (*CM*; the total duration of the infection is always 2 weeks); (B) the same as (A) but for total immune periods of ten years. In both cases avoiding infected individuals is detrimental for high infection rates for case mortalities up to 50% (and even greater, result not shown), but is always beneficial for diseases without recovery (*CM* = 1, light blue line). (C) Effect of the additional mortality induced by mild infections, for a case mortality of the classical disease of 10% and total immune periods of one year. For case mortalities of the mild infection (*CM_M_*; the total duration of the mild infection is always 2 weeks) below 5%, avoiding the pathogenic agent is detrimental for high infection rates. Above 5%, it is always beneficial.

**Figure 4:**
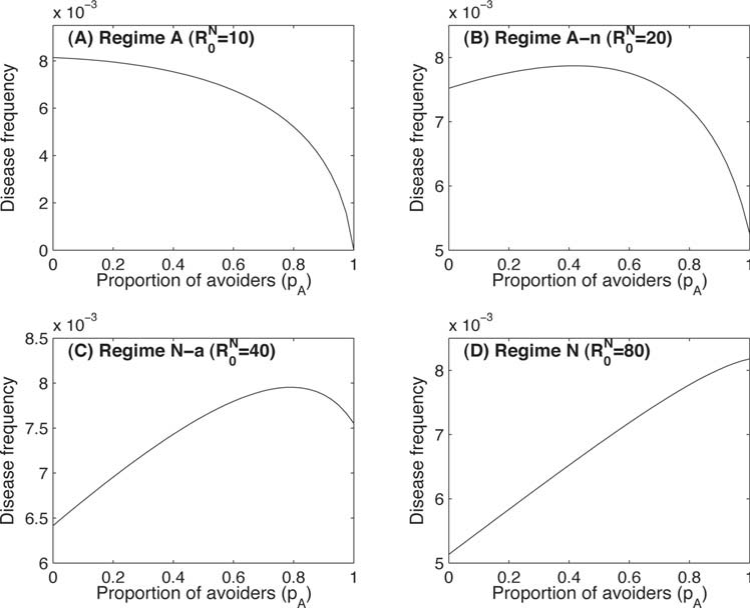
Impact of pathogen avoidance when the infection rate depends on the number of infected individuals in the population. We plot here the equilibrium proportion of infected individuals in the host population (Y-axis) according to the proportion of “avoiders” (*p_A_*, X-axis). Note that here the transmission rate of the pathogen can be derived from the value of 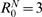 through the formula 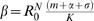. (A) An example of a situation where the system follows regime A (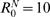, see Results: Model where exposure depends on infected individuals, for description of regimes); (B) an example of a situation where the system follows regime A-n (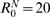); (C) an example of a situation where the system follows regime N-a (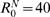) and (D) an example of a situation where the system follows regime N (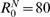).

**Figure 5:**
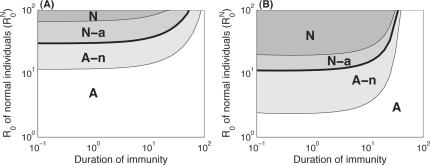
Effect of the parameters on the model where the infection rate depends on the number of infected individuals in the population. (A) Regime followed according to the basic reproductive number of normal individuals (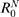) and duration of immunity (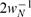); (B) same as (A) but with (*q* = ρ = 1). The bold lines represent the threshold value at equilibrium, where the number of infected individuals is the same in a population consisting only of “avoiders” and in a population exclusively composed of “normal” individuals.

In the first part of the analysis of the model with constant exposure and in the analysis of the model where the infection rate depends on infected individuals, we consider a pathogen that does not induce additional mortality on infected hosts (α = α*_M_* = 0, Fig. 2, 4 and 5). In the second part of the analysis of the model with constant exposure (Fig. 3), we consider the case of lethal pathogens, firstly only in their severe form (α>0, α*_M_* = 0, Fig. 3A, B) and then in both severe and mild forms (α>α*_M_*>0, Fig. 3C). It might seem surprising to assume additional mortality associated with mild infections. In fact, what we term a mild infection in our models is an infection acquired during a period of partial immunity. We use the term ‘mild infection’ because this kind of infection is generally mild. But these infections can also become severe (e.g. in individuals under stress), so it is natural to assume that these ‘mild infections’ may also increase the risk of death for individuals.

## Results

### Model with constant exposure rate

#### a) the case of non-lethal pathogens

First we focus on non-lethal infections (Fig. 2). In this case we look at the increase in disease frequency with pathogen avoidance, which is defined by the ratio of the frequency at which “avoiders” get infected compared to “normal” individuals. Note that an increase in disease frequency below a value of one means that avoiding the pathogen is in fact a beneficial strategy.

At a low infection rate, trying to avoid the infection is beneficial (Fig. 2A). In particular, it reduces the risk of developing the primary infection, whose severity cannot be reduced by the naïve immune system. In cases where the infection rate is very low, “avoiders” always contract classical disease ten times less frequently than “normal” individuals.

When the infection rate is high, a different scenario arises (see Fig. 2A). The primary infection occurs sooner or later, but “normal” individuals boost their immunity rapidly following the loss of their full immunity. So, they have little risk of developing the classical disease more than once in their lifetime. In contrast, avoiding infections increases the probability of losing partial disease protection, thereby increasing the risk of repeated development of the classical disease. However, at very high infection rates, even “avoiders” have little chance of losing partial disease protection and so the detrimental effect of avoiding the pathogen is less evident.

Another important parameter of the model is the total duration of acquired immunity (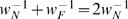 since we always have *w_N_* = *w_F_*) (Fig. 2B, C). The loss of partial immunity makes all individuals, and more especially “avoiders”, susceptible to repeated classical disease occurrences. Clearly, a longer immune period will reduce the number of secondary classical disease cases. With life-long immunity, secondary infections never occur and avoiding the pathogen is never a detrimental strategy (see Fig. 2B).

#### b) the case of lethal pathogens

Results for lethal pathogens are similar to those for non-lethal pathogens described above. But now we look at the decrease in life expectancy due to an avoidance strategy. Note that a negative decrease means that avoidance is in fact beneficial. Even for highly lethal pathogens, avoiding the pathogenic agent is always detrimental when infection rates are high. In fact, this detrimental effect becomes very small and occurs only for very high infection rates as soon as the case mortality is high (see for example Fig. 3A and *CM* = 0.5, red line). A longer immune period mitigates the detrimental effects of avoidance for pathogens with low case mortality (Fig. 3B).

The detrimental effect of pathogen avoidance remains true even when we assume additional mortality during mild infections (Fig. 3C), as long as the case mortality of the mild disease remains low (here below 5%). For example, with 1% case mortality for the mild disease, avoiding the pathogenic agent may reduce the life expectancy of individuals by 10 years.

Finally, it is important to note that in every case studied here (see Fig. 2 and Fig. 3), avoiding infections is always a good strategy when the infection rate is low.

#### Model where exposure depends on infected individuals

Now we explore the problem from a population perspective. We consider a population consisting of both “avoiders” and “normal” individuals (with a proportion *p_A_* of “avoiders”) and assume that the rate of infection of susceptible individuals depends on the number of each type of infected individuals (i.e. mildly infected and severely infected individuals). Our objective is to determine the effect of the proportion of “avoiders” (*p_A_*) on the impact of the disease. The idea is to simulate a public health measure and to determine which proportion of the population should avoid the infection to make the impact of the disease as low as possible. For the sake of simplicity, we assume no disease-induced mortality such that the impact of the disease is, here, related to the total number of infected individuals in the population.

We look at the disease frequency in the population at equilibrium as a function of the proportion of “avoiders” (*p_A_*) for different values of the basic reproductive number (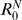) of the pathogen in a population consisting only of “normal” individuals (Fig. 4). In the following paragraphs the term “basic reproductive number of normal individuals” will be used to refer to 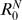.

We identify four regimes, depending on different parameter settings. In the first regime (Fig. 4A, regime A), the disease frequency at equilibrium is a decreasing function of the proportion of “avoiders” (*p_A_*). In this case the rate of infection is always too low for the “normal” strategy to be efficient. Avoiding infections is good for “avoiders”, but also for the population. If the proportion of individuals is such that the basic reproductive number of the pathogen is decreased below 1, then the pathogen goes extinct from the population. In the second regime (Fig. 4B, regime A-n), avoiding the infection can be beneficial, but only if a sufficient number of individuals in the population are “avoiders”. If only a small proportion of individuals avoid the infection, then the impact of the disease can be increased by increasing the number of “avoiders”. In contrast, for the third regime (Fig. 4C, regime N-a) the “normal” strategy is the best one, but if most of the individuals in the population are “avoiders” then increasing the proportion of “normal” individuals is deleterious. In the last regime (Fig. 4D, regime N), the number of infected individuals at equilibrium is an increasing function of the proportion of avoiders (*p_A_*). Even with all individuals avoiding the infection, the rate of infection cannot fall below the threshold where avoiding the infection becomes an efficient strategy, and so avoiding the infection is always deleterious. Note that in all regimes the best strategy is always an extreme one (*p_A_* = 0 or *p_A_* = 1).

Next, we investigate the effects of the basic reproductive number of “normal” individuals (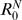) and of the total duration of immunity (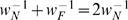 since *w_N_* = *w_F_*) (Fig. 5A). We look at the regime in which the system attains equilibrium. We find that for the smallest values of 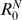 the system always follows the first regime (regime A), where avoiding infections is the best strategy and always reduces the impact of the pathogen. With increasing 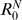 the system follows the second regime (regime A-n), where avoiding infection is still the best strategy, but can be detrimental if not enough individuals in the population are “avoiders”. Above a threshold (bold line in Fig. 5A) in 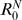, the system initially follows the third regime (regime N-a) and then regime N (the fourth regime). At this stage trying to make people avoid the infection is deleterious. With regard to the duration of immunity, we find the same effect as previously, i.e. avoiding infections is generally the best strategy for diseases inducing long immune memory.

Finally, we looked at what happens when we consider larger coefficients for mild re-infections (*q* = ρ = 1, Fig. 5B). From a qualitative perspective we find the same results. However, quantitatively, the threshold for 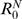, above which avoiding infections becomes a deleterious strategy, is largely reduced. Of course, for 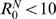, full avoidance is always the best strategy since it leads to eradication of the disease. But, for diseases inducing short-term immunity, as soon as 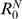 is slightly larger than 10, the ‘normal’ strategy becomes the most efficient one. Even when all individuals avoid the infection, so that the basic reproductive number of the pathogen is only slightly above 1, the impact of the disease cannot be reduced below its initial value (when all individuals are “normal”). Another interesting point is that even for small values of 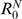 (here, for example, for 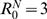) we observe situations where the system follows the second regime (regime A-n). This means that even for pathogens with low basic reproductive numbers, a minimal threshold in the proportion of “avoiders” (*p_A_*) must be attained to make the avoidance strategy beneficial.

## Discussion

Reducing pathogen exposure, e.g. by avoiding contact with infected individuals in the case of directly transmitted diseases or by avoiding vector bites in the case of vector-borne infections, is an attractive solution for reducing the impact of diseases. But this neglects the fact that when the immunity acquired against a pathogen must be boosted in order to remain efficient, the nature of the host-pathogen interaction depends on the intensity of exposure of the host [11]. In such circumstances, reducing exposure to pathogens provides both beneficial (because it decreases the probability of developing the severe disease for susceptible individuals) and detrimental (because it prevents boosts to immunity) effects. In such cost *versus* benefit trade-offs, mathematical models represent useful tools to assess the likelihood for success of different strategies in different contexts.

Avoiding exposure to the pathogenic agent changes the nature of the host-pathogen interaction, and can sometimes be a good strategy. This is the case for pathogens with limited transmissibility (e.g. sexually transmitted diseases such as gonorrhea), for pathogens inducing life-long immunity (e.g. measles) and, of course, for highly lethal diseases (e.g. HIV). The case of poorly transmissible pathogens can easily extend to all pathogens for which one may be exposed to a limited number of times during their lifetime. This is typically the case for travellers' diarrhoea, a disease acquired during visits to countries with low hygienic standards. For a traveller that visits such countries rarely, it is clearly better to avoid being infected.

In contrast, for highly transmissible pathogenic agents for which the immune response requires regular boosts to remain efficient, avoiding infections may be counter-productive and actually increases the deleterious impact of the pathogenic agent. For example, in the worst scenario modelled here, “avoiders” were more than six times more at risk of becoming sick compared to “normal” individuals (see Fig. 2B) or had a life-expectancy reduced by 30 years (see Fig. 3A). Many pathogenic agents that induce upper-respiratory tract infections (e.g. the common cold or influenza) or gastro-enteritis may fall into this category. They are often highly transmissible and/or induce short-term immunity. Influenza immunity also, to some extent, wanes gradually with time. Previous infection by a distinct strain may provide partial immunity against a new strain [16]–[18], provided that the antigenic distance between the two strains is not too high. As the virus continuously changes, the chance of encountering a strain that is partly recognized by the immune system decreases with the time since the previous infection.

Typically, most public health efforts have focused on reducing the frequency of exposure of individuals to pathogenic agents. This strategy, despite great successes, has limitations in certain circumstances. For example, limiting the circulation of a pathogen may change the age-specific incidence of the infection, sometimes leading to adverse effects in diseases for which age affects the clinical outcome of the infection [19]–[23]. Our work illustrates another aspect of a general concept that explains these relative failures. Defense against infections is not only a matter of avoiding contact with the pathogenic agent, but also of limiting the severity of infection.

Only an accurate knowledge of the factors influencing the severity of a disease may help determine if limiting the spread of the infectious agent is a good strategy. Today, the concept that regular exposure to pathogenic agents is crucial to maintain efficient immune defences against infectious diseases is widely accepted. However, little effort has been made to test this hypothesis. We believe that the framework we present here demonstrates that experimental studies must be enacted for diseases that need to be controlled. Such experiments, coupled with models like ours, would help with the assessment and design of control interventions.

## References

[1] Thomas SL, Wheeler JG, Hall AJ (2002). Contacts with varicella or with children and protection against herpes zoster in adults: a case-control study.. Lancet.

[2] Lu CY, Chiang BL, Chi WK, Chang MH, Ni YH (2004). Waning immunity to plasma-derived hepatitis B vaccine and the need for boosters 15 years after neonatal vaccination.. Hepatology.

[3] Wendelboe AM, Van Rie A, Salmaso S, Englund JA (2005). Duration of immunity against pertussis after natural infection or vaccination.. Pediatr Infect dis J.

[4] Narita M, Matsuzono Y, Takehoshi Y, Yamada S, Itakura O (1998). Analysis of mumps vaccine faillure by means of avidity testing for mumps virus-specific immunoglobuline G.. Clin Diagn Lab Immunol.

[5] Paunio M, Hedman K, Davidkin I, Valle M, Heinonen OP (2000). Secondary measles vaccine faillures identified by measurement of IgG avidity: high occurence among teenagers vaccinated at young age.. Epidemiol Infect.

[6] Moulton LH, Staat MA, Santosham M, Ward RL (1998). The protective effectiveness of natural rotavirus infection in an American Indian population.. J Infect Dis.

[7] Ausiello CM, Lande R, Urbani F, la Sala A, Stefanelli P (1999). Cell-Mediated Immune Responses in Four-Year-Old Children after Primary Immunization with Acellular Pertussis Vaccines.. Infect Immun.

[8] Leino T, Auranen K, Makela PH, Kayhty H, Takala AK (2000). Dynamics of natural immunity caused by subclinical infections, case study on Haemophilus influenzae type b (Hib).. Epidemiol Infect.

[9] Coulson BS, Grimwood K, Barnes GL, Bishop RF (1992). Role of coproantibody in clinical protection of children during reinfection with rotavirus.. J Clin Microbiol.

[10] Thelu J, Sheick-Zakiuddin I, Boudin C, Peyron F, Picot S (1991). Development of natural immunity in Plasmodium falciparum malaria: study of antibody response by Western immunoblotting.. J Clin Microbiol.

[11] Aguas R, Goncalves G, Gomes MG (2006). Pertussis: increasing disease as a consequence of reducing transmission.. Lancet Infect Dis.

[12] Gomes MG, White LJ, Medley GF (2005). The reinfection threshold.. J Theor Biol.

[13] Aguas R, White LJ, Snow RW, Gomes MG (2008). Prospects for malaria eradication in sub-Saharan Africa.. PLoS ONE.

[14] Snow RW, Marsh K (2002). The consequences of reducing transmission of Plasmodium falciparum in Africa.. Adv Parasitol.

[15] Snow RW, Omumbo JA, Lowe B, Molyneux CS, Obiero JO (1997). Relation between severe malaria morbidity in children and level of Plasmodium falciparum transmission in Africa.. Lancet.

[16] Boon AC, de Mutsert G, van Baarle D, Smith DJ, Lapedes AS (2004). Recognition of homo- and heterosubtypic variants of influenza A viruses by human CD8+ T lymphocytes.. J Immunol.

[17] Epstein SL (2006). Prior H1N1 influenza infection and susceptibility of Cleveland Family Study participants during the H2N2 pandemic of 1957: an experiment of nature.. J Infect Dis.

[18] Ferguson NM, Galvani AP, Bush RM (2003). Ecological and immunological determinants of influenza evolution.. Nature.

[19] Miller E, Gay N (1997). Effect of age on outcome and epidemiology of infectious diseases.. Biologicals.

[20] Fouchet D, Marchandeau S, Bahi-Jaber N, Pontier D (2007). The role of maternal antibodies in the emergence of severe diseases as a result of fragmentation.. J R Soc Interface.

[21] Bunimovich-Mendrazitsky S, Stone (2005). Modeling polio as a disease of development.. J Theor Biol.

[22] Zinkernagel RM (2001). Maternal antibodies, childhood infections, and autoimmune diseases.. N Engl J Med.

[23] Coleman PG, Perry BD, Woolhouse MEJ (2001). Endemic stability-a veterinary idea applied to human public health.. Lancet.

